# Guidelines for tuberculosis screening and preventive treatment among pregnant and breastfeeding women living with HIV in PEPFAR-supported countries

**DOI:** 10.1371/journal.pone.0296993

**Published:** 2024-04-16

**Authors:** Yael Hirsch-Moverman, Allison Hsu, Elaine J. Abrams, William P. Killam, Brittany Moore, Andrea A. Howard

**Affiliations:** 1 ICAP at Columbia University, New York, New York, United States of America; 2 Department of Epidemiology, Mailman School of Public Health Columbia University, New York, New York, United States of America; 3 Department of Pediatrics, Vagelos College of Physicians & Surgeons, Columbia University, New York, New York, United States of America; 4 Division of Global HIV and TB, U.S. Centers for Disease Control and Prevention, Atlanta, Georgia; Kisumu County, KENYA

## Abstract

**Background:**

Tuberculosis (TB) preventive treatment (TPT) is recommended by the World Health Organization (WHO) for persons living with HIV, including pregnant and breastfeeding women. Given the President’s Emergency Plan for AIDS Relief (PEPFAR)’s investment in TPT services for persons living with HIV as a strategy to prevent TB as well as uncertainty in guidelines and policy regarding use of TPT during pregnancy and the postpartum period, we conducted a review of current relevant national guidelines among PEPFAR-supported countries.

**Methods:**

Our review included 44/49 PEPFAR-supported countries to determine if TB screening and TPT are recommended specifically for pregnant and breastfeeding women living with HIV (WLHIV). National guidelines reviewed and abstracted included TB, HIV, prevention of vertical HIV transmission, TPT, and any other relevant guidelines. We abstracted information regarding TB screening, including screening tools and frequency; and TPT, including timing, regimen, frequency, and laboratory monitoring.

**Results:**

Of 44 PEPFAR-supported countries for which guidelines were reviewed, 66% were high TB incidence countries; 41% were classified by WHO as high TB burden countries, and 43% as high HIV-associated TB burden countries. We found that 64% (n = 28) of countries included TB screening recommendations for pregnant WLHIV in their national guidelines, and most (n = 35, 80%) countries recommend TPT for pregnant WLHIV. Fewer countries included recommendations for breastfeeding as compared to pregnant WLHIV, with only 32% (n = 14) mentioning TB screening and 45% (n = 20) specifically recommending TPT for this population; most of these recommend isoniazid-based TPT regimens for pregnant and breastfeeding WLHIV. However, several countries also recommend isoniazid combined with rifampicin (3RH) or rifapentine (3HP).

**Conclusions:**

Despite progress in the number of PEPFAR-supported countries that specifically include TB screening and TPT recommendations for pregnant and breastfeeding WLHIV in their national guidelines, many PEPFAR-supported countries still do not include specific screening and TPT recommendations for pregnant and breastfeeding WLHIV.

## Introduction

Tuberculosis (TB) is the leading cause of morbidity and mortality among persons living with HIV (PLHIV) who reside in low- and middle-income countries (LMIC) with a high TB burden [1]. Among women, TB incidence peaks during reproductive age, irrespective of HIV status. The World Health Organization (WHO) reports incidence rate ratios of TB in pregnant and postpartum women as compared to non-pregnant women of 1.4 and 1.9, respectively [2–5]. The End TB Strategy calls for provision of TB preventive treatment (TPT) for persons at high risk for TB [6, 7]. Studies have shown that provision of TPT to PLHIV reduced TB incidence and mortality by 37%, independent of antiretroviral therapy (ART) status [8, 9].

Of the 38.4 million PLHIV, 19.7 million are women aged ≥15 years, and an estimated 1.3 million women living with HIV (WLHIV) became pregnant in 2021 [10–12]. Both HIV and pregnancy are associated with immune suppression and an increased risk of progression to active TB disease [13–15]. Maternal TB disease can have devastating consequences for both mothers and infants including increased maternal and infant mortality; adverse pregnancy outcomes including prematurity, low birth weight, and intrauterine growth restriction; and infant TB [5, 16–20]. TB in pregnant women increases the risk of maternal mortality three-fold (odds ratio [OR] = 2.8, 95% confidence interval [CI] 1.7–4.6) and perinatal death four-fold (OR = 4.2; 95% CI 1.5–11.8) [20]. Infants infected with TB perinatally have a very high risk of death [18, 19]. Pregnant WLHIV have a high risk of acquiring TB, which can have severe consequences for both mother and infant [17]. Maternal TB more than doubles the risk of perinatal HIV transmission and significantly increases mortality for all children in the household [17, 21–24].

The 2011 WHO guidelines on isoniazid preventive therapy (IPT) for PLHIV in resource-constrained settings recommend IPT for all PLHIV regardless of pregnancy status [7]. However, these guidelines as well as the 2018 WHO guidelines on latent TB infection advise caution and clinical judgement when deciding the best time to start TPT in pregnant women in light of emerging safety concerns of TPT during pregnancy [25]. The International Maternal Pediatric Adolescent AIDS Clinical Trials Network (IMPAACT) P1078 TB APPRISE study, a phase 4 randomized controlled trial evaluating the safety and timing of IPT initiation among pregnant WLHIV in high TB burden settings, reported no difference in maternal hepatotoxicity but found a higher risk of a composite adverse pregnancy outcome (fetal demise, prematurity, low birth weight, and congenital anomaly) among women who received IPT during pregnancy compared to those who delayed IPT initiation until the postpartum period [26]. However, observational sub-studies of TPT trials assessing the use of daily and weekly isoniazid during pregnancy or at conception found no association between IPT use and adverse pregnancy outcomes [27, 28]. A recent systematic review and meta-analysis of nine studies found inconsistent associations between IPT and adverse pregnancy outcomes [27, 28]. The authors concluded that considering the grave consequences of active TB in pregnancy, current evidence does not support systematic deferral of IPT until the postpartum period [29]. This conclusion was echoed in the 2020 WHO Consolidated Guidelines on Tuberculosis module on prevention, which strongly encouraged, when feasible, baseline liver function tests (LFTs) when IPT is given in pregnancy or within three months of delivery [30]. No specific guidance on TPT for breastfeeding women was provided despite countries recommending deferral of TPT to the postpartum period, which may raise concerns for providers and patients about the infant’s exposure to TPT drugs through breast milk.

Implementation and scale-up of TB case finding, prevention, and treatment services have been a major focus in the U.S. President’s Emergency Plan for AIDS Relief (PEPFAR) programming. In 2018, the Department of State’s Office of the U.S. Global AIDS Coordinator and Health Diplomacy announced a goal to provide TPT to all 13.6 million PLHIV who are on ART in PEPFAR-supported countries by 2021. In 2019, the Department of State’s Office of the U.S. Global AIDS Coordinator and Health Diplomacy included provision of TPT for all PLHIV as one of 13 minimum program requirements for PEPFAR funding [31]. The Technical Considerations in the PEPFAR 2022 Country and Regional Operational Plan (COP/ROP) Guidance for all PEPFAR-Supported Countries stated that it is imperative for PMTCT programs to screen for active TB during clinical encounters and ensure linkage to diagnostic testing and treatment, however given the uncertainties around the safety, efficacy and appropriate timing of TPT in pregnant WLHIV, country programs should consider the benefits and risks of deferring TPT initiation for pregnant WLHIV based on their ART history, clinical presentation, and documentation of close contact with a person with active TB disease [32]. More specific guidance for breastfeeding WLHIV was not provided.

Given PEPFAR’s investment in TPT services for PLHIV, and the lack of clarity in guidelines and policy regarding use of TPT during pregnancy or breastfeeding, we conducted a review of current relevant national TB and HIV guidelines among PEPFAR-supported countries. Our objectives were to determine: (1) if TB screening is currently recommended specifically for pregnant and breastfeeding WLHIV; (2) whether TPT is currently recommended during pregnancy or the postpartum period; and (3) whether any laboratory or radiologic tests are recommended when evaluating WLHIV for TPT eligibility during pregnancy or the postpartum period.

## Materials and methods

We obtained current national guidelines from countries supported by PEPFAR through CDC in 2021 (n = 49), including TB, HIV, prevention of maternal-to-child HIV transmission (PMTCT), TPT, and any other relevant guidelines in these countries. Guidelines were collected between November 2021 and June 2022.

We created a data abstraction form to record information on recommendations regarding: TB screening, including screening tools and frequency; and TPT, including timing, regimen, frequency, and laboratory testing for eligibility and monitoring, for the following populations: (1) PLHIV, (2) pregnant WLHIV, and (3) breastfeeding WLHIV. The data abstraction form was piloted and modified to ensure consistency and clarity.

Data abstraction was conducted between December 2021 and June 2022, with the exception of the updated South African 2022 TPT guidelines, which were published and abstracted in September 2022 and the Vietnam and Thailand guidelines, which were abstracted in December 2022. Two trained data abstractors fluent in the language of the guidelines abstracted information from all guideline documents for a given country independently except for Thailand where the abstraction was conducted by a single data abstractor. The two abstractors then met to discuss their findings and resolve any discrepancies. Where discrepancies could not be resolved, an investigator was consulted.

Abstracted data for each country were compiled into a comprehensive table containing key information for each country. This table contained information for PLHIV, pregnant WLHIV, and breastfeeding WLHIV and was used to create a dashboard with filtering capabilities that allowed for a quick visual summary of recommendations [33]. In instances where a country had multiple guidelines and TB screening was recommended for a specific population in one guideline and another guideline did not include this information, the country was recorded as “TB screening recommended.” The same rule was applied to TPT recommendations. In the one instance where the guidelines for a particular country were contradictory, (i.e., TPT was recommended for a population in one guideline and not recommended in another guideline), we used the guideline that was published more recently to categorize the country. We defined high TB incidence countries as >100 TB cases per 100,000 population reported in 2020, and defined countries as high TB, TB/HIV or MDR/RR-TB burden based on WHO’s classification [1, 34].

## Results

We obtained and reviewed guidelines from 90% (44/49) of PEPFAR-supported countries. Both TB and HIV guidelines were reviewed for all countries except Cambodia and Senegal, where we only obtained HIV guidelines. Some countries also had PMTCT and/or TPT guidelines available for review. Guideline publication year varied greatly within and across countries, with Haiti having the oldest guideline (TB guidelines from 2009) and Ukraine and South Africa having the most recent guidelines in place (PMTCT and HIV guidelines from 2022, TPT guidelines from 2022, respectively).

Of the 44 countries for which guidelines were reviewed, 66% (29/44) were high TB incidence countries; 18 countries (41%) were classified by WHO as high TB burden countries, 19 (43%) were classified as high HIV-associated TB burden countries, and 14 (32%) were classified as high multi-drug resistant/rifampicin-resistant TB (MDR/RR-TB) burden countries [1]. More than half (27/44) of countries were from the WHO African Region (see Table 1).

**Table 1:** Number of countries classified by WHO as high TB incidence, high TB burden, high TB/HIV burden, or high MDR/RR-TB burden, by WHO region.

	Number of countries	High TB incidence*	High TB burden^#^	High TB/HIV burden^#^	High MDR/RR-TB burden^#^
African Region	27	22	13	15	7
Region of the Americas	8	1	1	1	0
European Region	4	1	0	0	4
South-East Asian Region	4	4	4	3	3
Western Pacific Region	1	1	0	0	0
Total	44	29	18	19	14

*>100 TB cases /100,000 population reported in 2020 [1]

^#^as defined by WHO [34]

MDR-TB = multidrug resistant TB, RR-TB = rifampicin resistant TB, TB = tuberculosis, WHO = World Health Organization

**Fig 1:**
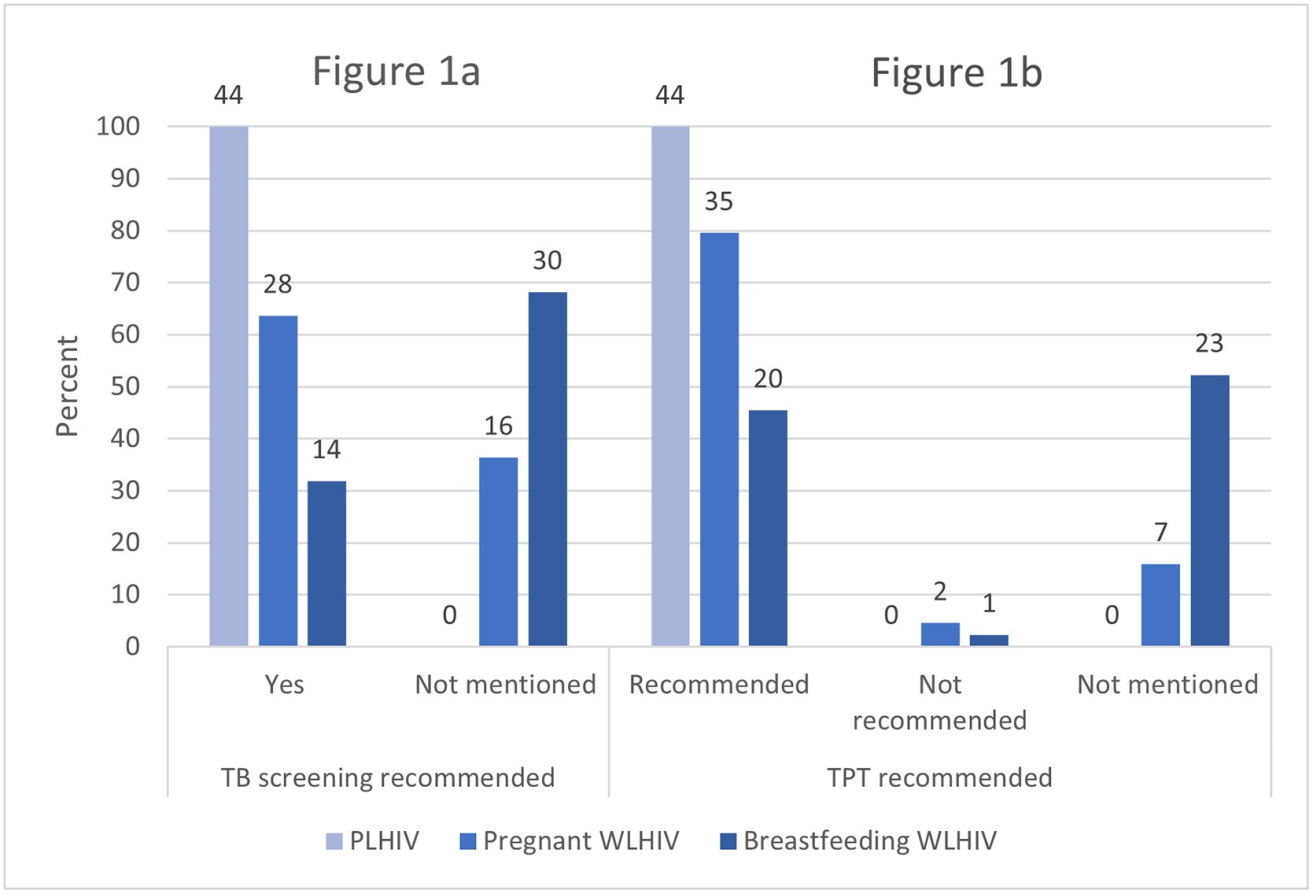
TB screening and TPT recommendations for people living with HIV overall, pregnant women living with HIV, and breastfeeding women living with HIV in national guidelines for PEPFAR-supported countries (n = 44).

As shown in Fig 1, TB screening and TPT recommendations for PLHIV were included in guidelines from all 44 countries reviewed. With regards to pregnant WLHIV, we found that 64% (n = 28) of countries included recommendations for TB screening in at least one of their national guidelines (Fig 1a) and 84% (n = 37) included recommendations regarding TPT, with 80% (n = 35) recommending TPT for pregnant WLHIV and 5% (n = 2) not recommending TPT for pregnant WLHIV (Fig 1b). Fewer countries had specific recommendations for breastfeeding WLHIV in their national guidelines, with only 32% (n = 14) of countries including TB screening recommendations (Fig 1a) and 48% (n = 21) including TPT recommendations, with 45% (n = 20) recommending TPT and 2% (n = 1) not recommending TPT for breastfeeding women (Fig 1b).

S1 Table summarizes guidelines for all countries reviewed, outlining TB screening and TPT recommendations, TPT regimens, and any special considerations, with a focus on pregnant and breastfeeding WLHIV.

### Recommendations for pregnant women living with HIV

In all 28 countries that specifically mention TB screening for pregnant WLHIV in their guidelines, a symptom screen is recommended; however, five of these countries (Brazil, Kenya, Nigeria, Thailand, Zimbabwe) also recommend a chest x-ray. Zimbabwe additionally recommends using body mass index as a TB screening tool for pregnant WLHIV and a molecular WHO-recommended rapid diagnostic test (mWRD) for individuals newly diagnosed with HIV in their 2017 TB guidelines. South Africa included a recommendation that pregnant WLHIV receive a mWRD at the first antenatal care visit in their 2022 TPT guidelines.

TPT during pregnancy is recommended in guidelines from 36 countries; for five of these countries, the guidelines did not specify a TPT regimen. Of the 31 countries that specify a TPT regimen for pregnant WLHIV, all recommend an isoniazid-based regimen. Among these, four countries also recommend an alternate regimen: Lesotho only specifies three months of isoniazid and rifampicin (3RH) in their 2021 PMTCT guidelines, while in their older guidelines six months of isoniazid (6H) is recommended; Nigeria specifies 3RH as an alternative to 6H and nine months of isoniazid (9H) in their 2020 HIV guidelines; Uganda recommends three months of isoniazid and rifapentine (3HP) as an equally viable alternative to 6H if the patient is not on protease inhibitors in their 2020 HIV guidelines; and Zimbabwe recommends 3HP over 6H –the 2020 TPT guidelines only recommend 6H if contraindications to 3HP exist. For Burundi, TPT for pregnant WLHIV is not mentioned in the 2020 HIV guidelines. However, in the 2020 TB guidelines, TPT is not recommended for pregnant WLHIV, stating that pregnant women should defer TPT until the postpartum period. Malawi’s 2018 HIV guidelines recommend TPT for pregnant WLHIV, but this recommendation was reversed in their 2019 addendum to the 2018 HIV guidelines, where it is stated that the TPT regimens recommended in Malawi (6H and 3HP) are not suitable for pregnant WLHIV. In Uganda, the 2020 HIV guidelines state that pregnant WLHIV should defer TPT until three months post-delivery unless there is a history of TB exposure or advanced HIV disease (WHO Stage 3/4 or CD4 count <200 cells/mm^3^), in which case either 6H or 3HP are recommended. South Africa also conditionally recommends deferring TPT until 6 weeks after delivery for pregnant WLHIV with a CD4 count >350 cells/mm^3^ in their 2020 HIV guidelines and 2022 TPT guidelines. The 2021 HIV guidelines from Thailand recommend TPT if the pregnant WLHIV was exposed to TB within the last year, otherwise it is recommended to defer TPT until 12 weeks postpartum.

LFTs prior to TPT initiation are required for pregnant WLHIV in three country guidelines: Cambodia (HIV 2020), Vietnam (TPT 2021), and Zambia (TPT 2019); Vietnam also requires monthly LFTs during TPT. Five other country guidelines strongly encourage baseline LFTs in pregnant WLHIV: Eswatini (TB 2019), India (TPT 2021), Kenya (TB 2021), Uganda (HIV 2020), and Zimbabwe (TB 2017). Uganda recommends doing LFTs both at baseline and after three months of treatment if possible, as well as closely monitoring WLHIV who get pregnant while on TPT for side effects.

#### Recommendations for breastfeeding women living with HIV

All 14 countries that mention TB screening for breastfeeding WLHIV in their national guidelines recommend a symptom screen; none recommend other TB screening tools specifically for breastfeeding WLHIV.

Only 20 countries specifically recommend TPT for breastfeeding WLHIV, including 18 countries that specify the TPT regimen and two countries that do not specify the regimen (Nigeria and Zambia). All regimens recommended for breastfeeding WLHIV are isoniazid-based, except Thailand, where only 3HP is recommended in their 2021 HIV guidelines. Eswatini includes 3HP as an equally viable alternative to 6H as long as ART medications are not contraindicated in their 2019 TB guidelines. Eswatini also recommends that TPT be initiated after one month of ART in both their 2019 TB guidelines and 2018 HIV guidelines, and within three months of delivery in their 2019 TB guidelines. Burundi recommends the postponement of TPT until the cessation of breastfeeding in their 2020 TB guidelines. The Thailand 2021 HIV guidelines recommend that unless the breastfeeding woman was recently exposed to TB (i.e., in the past year), TPT should be deferred until 12 weeks postpartum.

LFTs prior to TPT initiation in breastfeeding WLHIV are required in three country guidelines: Cambodia (HIV 2020), Vietnam (TPT 2021), and Zambia (TPT 2019); Vietnam also requires monthly LFTs during TPT. Three other country guidelines strongly encourage baseline LFTs: Eswatini (TB 2019), India (TPT 2021), and Zimbabwe (TB 2017); Zimbabwe guidelines refer specifically to the period within 3 months of delivery.

#### Recommendations in high vs. low TB burden countries

Table 2 illustrates the differences in TB screening and TPT recommendations for pregnant and breastfeeding WLHIV between high and low TB burden countries. Countries classified as high TB incidence, high TB burden and/or high TB/HIV burden are more likely to include recommendations on TB screening and TPT specifically for pregnant and breastfeeding WLHIV in their national guidelines.

**Table 2:** Comparison of inclusion of TB screening and TPT recommendations for pregnant and breastfeeding women living with HIV in national guidelines in high versus low TB incidence, TB burden, and TB/HIV burden countries.

	High				Low			
**TB incidence**			N = 29				N = 15		
	Pregnant WLHIV	TB screening recommended	21	72%	Pregnant WLHIV	TB screening recommended	7	47%	
		TPT recommended	25	86%		TPT recommended	10	67%	
		TPT regimen specified	22	76%		TPT regimen specified	8	53%	
	Breastfeeding WLHIV	TB screening recommended	11	38%	Breastfeeding WLHIV	TB screening recommended	3	20%	
		TPT recommended	17	59%		TPT recommended	3	20%	
		TPT regimen specified	15	52%		TPT regimen specified	3	20%	
**TB burden**			N = 18				N = 26		
	Pregnant WLHIV	TB screening recommended	16	89%	Pregnant WLHIV	TB screening recommended	12	46%	
		TPT recommended	18	100%		TPT recommended	17	65%	
		TPT regimen specified	16	89%		TPT regimen specified	14	54%	
	Breastfeeding WLHIV	TB screening recommended	8	44%	Breastfeeding WLHIV	TB screening recommended	6	23%	
		TPT recommended	13	72%		TPT recommended	7	27%	
		TPT regimen specified	11	61%		TPT regimen specified	7	27%	
**TB/HIV burden**			N = 19				N = 25		
	Pregnant WLHIV	TB screening recommended	18	95%	Pregnant WLHIV	TB screening recommended	10	40%	
		TPT recommended	18	95%		TPT recommended	17	68%	
		TPT regimen specified	16	84%		TPT regimen specified	14	56%	
	Breastfeeding WLHIV	TB screening recommended	10	53%	Breastfeeding WLHIV	TB screening recommended	4	16%	
		TPT recommended	13	68%		TPT recommended	7	28%	
		TPT regimen specified	11	58%		TPT regimen specified	7	28%	

## Discussion

In this review we found that nearly two thirds of PEPFAR-supported countries include TB screening recommendations for pregnant WLHIV in their national guidelines, while the remainder do not specifically address pregnancy in their TB screening guidelines for PLHIV. While most (80%) countries recommend TPT for pregnant WLHIV, two countries (Burundi and Malawi) recommend not administering TPT during pregnancy, and three countries (South Africa, Thailand, and Uganda) only recommend TPT for pregnant WLHIV in specific circumstances that place them at increased risk for TB. Fewer PEPFAR-supported countries include recommendations for breastfeeding WLHIV in their national guidelines, with less than a third (32%) mentioning TB screening tools and less than half (45%) specifically recommending TPT for this population. One country (Burundi) recommends not administering TPT until the cessation of breastfeeding, and one country (Thailand) only recommends TPT for breastfeeding WLHIV in specific circumstances that place them at increased risk for TB. Eight countries require or recommend LFTs prior to TPT during pregnancy, while six countries recommend baseline LFTs during the postpartum period.

A review of TB screening and TPT recommendations for pregnant WLHIV in national guidelines published before 2017 from 38 high TB and TB/HIV burden countries found a lower proportion of countries that included TB screening (39%) and TPT recommendations (64%) than was found in our review [35]. Over half (22/38) of the included countries in this recent review overlapped with countries included in our review. Our findings suggest that there has been an increase in the proportion of high TB and TB/HIV burden countries that include TB screening and TPT recommendations for pregnant WLHIV in their national guidelines since 2017 [35]. This increase may be attributable to promotion of the End TB Strategy by the United Nations, WHO, and PEPFAR, which includes collaborative TB/HIV activities and increasing TPT provision for persons at high risk, as well as the Department of State’s Office of the U.S. Global AIDS Coordinator and Health Diplomacy’s inclusion of TPT provision for all PLHIV as a minimum program requirement for PEPFAR funding [6, 31]. Despite the apparent increase in the proportion of high TB and TB/HIV burden countries that include TB screening and TPT recommendations for pregnant WLHIV in their national guidelines, this is not evident in low TB and TB/HIV burden countries that receive PEPFAR support, where there is a substantially lower proportion of countries that include specific recommendations on TB screening or TPT for pregnant WLHIV. This could be because countries with a higher burden of disease have a larger population at risk for TB and a need for clear guidance to be able to meet WHO TPT targets in the End TB Strategy.

To our knowledge, this is the first report that summarizes TB screening and TPT recommendations for breastfeeding WLHIV in national guidelines. Although WHO in 2011 recommended TPT for pregnant and breastfeeding women, we found that only 45% of PEPFAR-supported countries specifically recommend TPT for breastfeeding women, which is particularly relevant given that for many WLHIV, TPT will have been deferred during pregnancy. It is imperative that country guidelines routinely indicate whether their recommendations for adults apply to breastfeeding WLHIV and provide specific recommendations for this population when indicated. The postpartum period potentially represents an important time when women are still in care and should be receiving TPT that was postponed during pregnancy, and yet it is also a period when providers and patients may have concerns about the infant’s exposure to TPT drugs through breast milk.

While most countries recommend isoniazid-only TPT regimens for pregnant and breastfeeding WLHIV, several also recommend isoniazid combined with rifampicin or rifapentine. Current WHO guidelines suggest that more evidence is needed regarding use of rifamycins, and in particular rifapentine, in pregnant WLHIV even if pregnancy should not disqualify WLHIV from receiving 3RH [30]. Nigeria and Lesotho recommend 3RH along with isoniazid-based regimens in pregnant WLHIV. 3HP is currently not recommended during pregnancy by the CDC owing to limited data on rifapentine pharmacokinetics, dosing, and safety during pregnancy [25, 30, 36]. More data is needed on the rifamycin agents as well as new agents being studied for TPT in nonpregnant adults [37]. Despite this, both Zimbabwe and Uganda recommend 3HP in addition to isoniazid-based regimens in pregnant WLHIV, and both Thailand and Eswatini recommend 3HP in breastfeeding WLHIV. IMPAACT 2001, which studied the pharmacokinetics and safety of 3HP among pregnant women with indications for TPT, found no drug-related serious adverse events [38]. The authors concluded that 3HP use in pregnant women was safe and tolerable. There is an urgent need for timely safety and pharmacokinetic studies of new TPT regimens during the pregnancy and breastfeeding periods, and for strong pharmacovigilance systems and targeted cohort studies to further assess safety in pregnancy and breastfeeding without delaying drug access to women [37–40].

The routine exclusion of pregnant and breastfeeding women from clinical trials of new treatments, including treatment for TB, results in no clear dosing information and safety considerations when the drug is approved and moved into programmatic settings. Additionally, because birth and pregnancy outcomes are rarely studied in controlled trials, resulting data are difficult to interpret. These factors may be contributing to countries’ hesitancy to include TPT recommendations for these vulnerable populations in their guidelines.

Because of the evolving science, WHO frequently updates its TPT recommendations, and countries must in turn revise their national guidelines to incorporate the latest WHO recommendations. The importance of this review is that it provides a summary of current TB screening and TPT recommendations for pregnant and breastfeeding WLHIV, two highly vulnerable groups, specifically comparing high and low TB and TB/HIV burden countries in different regions of the globe that receive PEPFAR support. The accompanying dashboard allows for a quick understanding of the gaps in TB screening and TPT policy for pregnant and breastfeeding WLHIV compared to PLHIV overall [33].

A limitation of our review is that there are some inconsistencies among guidelines from the same country, whereby the TB screening and/or TPT recommendations for pregnant and breastfeeding WLHIV in the TB guidelines may differ from those in the HIV guidelines. This is most likely a reflection of changes in WHO guidance and the time it takes to update national guidelines. Another limitation was that we were unable to identify publicly available guidelines for all countries supported by PEPFAR through CDC, and did not include four countries supported by PEPFAR through another U.S. government agency. Despite these limiting factors, we were able to include most of the original list of countries in our review (44/49, 90%). It is important to note that our findings do not necessarily reflect the extent to which pregnant and breastfeeding WLHIV have access to TB screening and TPT services in PEPFAR-supported countries, as recommendations for “all PLHIV” might be relevant in some countries. While guidelines are important to inform policy, there is often a long lag between guideline development and implementation in the field. A strength of our review is that we included data from the most recent guidelines that were available during our data collection period from a wide geographic area. Additionally, we abstracted and analyzed data specific to breastfeeding WLHIV, a population that is not usually disaggregated from the general population of PLHIV, as well as abstracted data regarding guidance on obtaining LFTs prior to TPT during pregnancy and the postpartum period.

## Conclusions

Despite progress in the number of PEPFAR-supported countries that specifically include TB screening and TPT recommendations for pregnant and breastfeeding WLHIV in their national guidelines, many PEPFAR-supported countries still do not provide screening or TPT recommendations specifically for pregnant and breastfeeding WLHIV or provide guidance on the operationalization and implementation of these guidelines in programmatic settings. This may impact WLHIV’s access to these potentially life-saving services.

## Supporting information

S1 Table TB screening and TPT recommendations for pregnant and breastfeeding women living with HIV, listed alphabetically within WHO region. (DOCX)
