# Supplementary material for: Guidelines for tuberculosis screening and preventive treatment among pregnant and breastfeeding women living with HIV in PEPFAR-supported countries
Source: PLoS One. 2024 Apr 16;19(4):e0296993. doi: 10.1371/journal.pone.0296993 (PMC11021021; doi:10.1371/journal.pone.0296993)
Supplement: S1 Table — (DOCX) [file pone.0296993.s001.docx]

**S1 Table: TB screening and TPT recommendations for pregnant and breastfeeding women living with HIV, listed alphabetically within WHO region**

| **Country** | **Number of guidelines reviewed** | **Year of guidelines** | **Pregnant WLHIV** | | | | **Breastfeeding WLHIV** | | | |
| --- | --- | --- | --- | --- | --- | --- | --- | --- | --- | --- |
|  |  |  | **TB screening recommended** | **TPT recommended** | **TPT regimen** | **Special considerations** | **TB screening recommended** | **TPT recommended** | **TPT regimen** | **Special considerations** |
| **African Region** | | | | | | | | | | |
| **Angola** | 2 | 2018, 2020 | Yes | Yes | 6H | N/A | No mention | No mention | No mention | N/A |
| Benin | 2 | 2019 | No mention | No mention | No mention | N/A | No mention | No mention | No mention | N/A |
| Burkina Faso | 2 | 2019, 2021 | No mention | No mention | No mention | N/A | No mention | No mention | No mention | N/A |
| **Burundi** | 2 | 2020 | No mention | No | N/A | Defer until postpartum | No mention | No | No mention | Defer until cessation of breastfeeding |
| **Cameroon** | 2 | 2019 | Yes | Yes | 6H | N/A | No mention | Yes | 6H | N/A |
| **Cote d’Ivoire** | 4 | 2018, 2020, 2021 (x2) | No mention | No mention | No mention | N/A | No mention | No mention | No mention | N/A |
| **Democratic Republic of the Congo** | 3 | 2020, 2021 | Yes | Yes | 6H | N/A | No mention | Yes | 6H | N/A |
| **Eswatini** | 2 | 2018, 2019 | Yes | Yes | 6H | Start after 1m of ART, baseline LFT recommended (2019) | Yes | Yes | 6H, 3HP | Start within 3m of delivery and after 1m of ART; baseline LFT recommended (2019) |
| **Ethiopia** | 3 | 2018, 2020 (x2) | Yes | Yes | 6H | N/A | Yes | No mention | No mention | N/A |
| **Ghana** | 2 | 2019 (x2) | Yes | Yes | No mention | N/A | No mention | No mention | No mention | N/A |
| **Kenya** | 3 | 2018, 2020, 2021 | Yes | Yes | 6H | Baseline LFT recommended | No mention | Yes | 6H | N/A |
| **Lesotho** | 3 | 2016, 2019, 2021 | Yes | Yes | 6H, 3RH | N/A | Yes | Yes | 6H | N/A |
| **Malawi** | 2 | 2018 (x2), 2019 | Yes | No | N/A | N/A | No mention | No mention | No mention | N/A |
| Mali | 2 | 2020, 2021 | Yes | Yes | 6H | N/A | Yes | Yes | 6H | N/A |
| **Mozambique** | 4 | 2014, 2016, 2019, 2020 | Yes | Yes | 6H | Initiate after 2w of ART/CTX (2014) | Yes | Yes | 6H | Initiate after 2w of ART/CTX (2014) |
| **Namibia** | 3 | 2019 (x2), 2021 | Yes | Yes | 6H, 9H | 3HP not to be used | No mention | No mention | No mention | N/A |
| **Nigeria** | 4 | 2019, 2020 (x2), 2021 | Yes | Yes | 6H, 9H, 3RH | N/A | Yes | Yes | No mention | N/A |
| Rwanda | 2 | 2020 (x2) | No mention | No mention | No mention | N/A | Yes | No mention | No mention | N/A |
| **Senegal** | 2 | 2019, unknown | No mention | No mention | No mention | N/A | No mention | No mention | No mention | N/A |
| **Sierra Leone** | 2 | 2018, 2020 | Yes | Yes | 6H | N/A | No mention | Yes | 6H | N/A |
| **South Africa** | 3 | 2014, 2020  2022 | Yes | Yes, conditionally | 6H, 12H | Defer TPT until 6w after delivery if CD4>350 cells/uL (2020, 2022) | Yes | Yes | 12H | N/A |
| **South Sudan** | 4 | 2016, 2019, 2020, 2021 | Yes | Yes | 6H | Can be postponed until after delivery (2021) | Yes | Yes | 6H | N/A |
| **Tanzania** | 2 | 2019, 2020 | Yes | Yes | 6H | N/A | Yes | Yes | 6H | N/A |
| Togo | 2 | 2019, 2020 | Yes | No mention | No mention | N/A | No mention | No mention | No mention | N/A |
| **Uganda** | 2 | 2019, 2020 | Yes | Yes, conditionally | 6H, 3HP | Defer until 3m post-delivery unless history of exposure or advanced HIV disease, LFT at baseline & 3m recommended (2020) | Yes | No mention | No mention | N/A |
| **Zambia** | 3 | 2017, 2019, 2020 | Yes | Yes | No mention | Postpone until after first trimester; baseline LFT required (2019) | Yes | Yes | No mention | Baseline LFT required (2019) |
| **Zimbabwe** | 3 | 2016, 2017, 2020 | Yes | Yes | 6H, 3HP | Baseline LFT recommended (2017) | Yes | No mention | No mention | Baseline LFT recommended if within 3 months of delivery (2017) |
| **Region of the Americas** | | | | | | | | | | |
| Brazil | 3 | 2018, 2019, 2021 | Yes | Yes | 6H, 9H | N/A | No mention | No mention | No mention | N/A |
| Dominican Republic | 2 | 2018, 2020 | Yes | Yes | 6H | N/A | No mention | No mention | No mention | N/A |
| El Salvador | 2 | 2016, 2020 | No mention | No mention | No mention | N/A | No mention | No mention | No mention | N/A |
| Guatemala | 2 | 2018, 2019 | Yes | Yes | 6H | N/A | No mention | No mention | No mention | N/A |
| **Haiti** | 2 | 2009, 2016 | No mention | Yes | 36H | N/A | No mention | No mention | No mention | N/A |
| Honduras | 2 | 2018, 2020 | No mention | Yes | 9H | N/A | No mention | Yes | 9H | N/A |
| Nicaragua | 2 | 2010, 2016 | No mention | Yes | 6H | N/A | No mention | No mention | No mention | N/A |
| Panama | 2 | 2016, 2017 | Yes | Yes | 6H | N/A | Yes | No mention | No mention | N/A |
| **European Region** | | | | | | | | | | |
| Kazakhstan | 3 | 2019, 2020 (x2) | No mention | Yes | No mention | N/A | No mention | No mention | No mention | N/A |
| **Kyrgyz Republic** | 2 | 2014, 2020 | No mention | Yes | 6H | N/A | No mention | No mention | No mention | N/A |
| Tajikistan | 2 | 2018, 2019 | No mention | Yes | No mention | N/A | No mention | No mention | No mention | N/A |
| Ukraine | 3 | 2021, 2022 (x2) | Yes | Yes | 6H | N/A | No mention | Yes | 6H | N/A |
| **South-East Asian Region** | | | | | | | | | | |
| **India** | 3 | 2016, 2018, 2021 | Yes | Yes | 6H | Baseline LFT recommended (2021) | No mention | Yes | 6H | Baseline LFT recommended (2021) |
| **Myanmar** | 3 | 2018, 2020 (x2) | No mention | Yes | 6H | HP not to be used (2020) | No mention | Yes | 6H | N/A |
| **Thailand** | 2 | 2021 (x2) | Yes | Yes, conditionally | No mention | TPT recommended if recent TB exposure (within 1 yr) otherwise defer to 12w postpartum;  RFP not indicated in pregnant women | No mention | Yes | 3HP | Defer to 12w postpartum |
| **Vietnam** | 3 | 2019, 2020, 2021 | No mention | Yes | 9H | Monthly LFT (2021), 3HP contraindicated | No mention | Yes | 9H | Monthly LFT (2021) |
| **Western Pacific Region** | | | | | | | | | | |
| **Cambodia** | 1 | 2020 | No mention | Yes | 6H | Baseline LFT required | No mention | Yes | 6H | Baseline LFT required |

**Bold font** indicates high TB incidence (>100 TB cases/100,000 population reported in 20210; WLHIV= women living with HIV; LFT = liver function tests; CTX = cotrimoxazole; 6H = 6 months of daily isoniazid; 9H = 9 months of daily isoniazid; 12H = 12 months of daily isoniazid; 36H = 36 months of daily isoniazid/lifetime treatment; 3RH = 3 months of daily isoniazid + rifampicin; 3HP = 3 months of weekly isoniazid + rifapentine
